# Coexistence of Cor Triatriatum Sinister, Fibroelastoma and Pulmonary Veins Ostial Anatomy Variant as Incidental Findings in Coronary Computed Tomography Angiography

**DOI:** 10.3390/diagnostics12061449

**Published:** 2022-06-13

**Authors:** Paweł Gać, Adrian Martuszewski, Patrycja Paluszkiewicz, Rafał Poręba

**Affiliations:** 1 Centre for Diagnostic Imaging, 4th Military Hospital, Weigla 5, PL 50-981 Wroclaw, Poland; 2 Department of Population Health, Division of Environmental Health and Occupational Medicine, Wroclaw Medical University, Mikulicza-Radeckiego 7, PL 50-368 Wroclaw, Poland; adrian.martuszewski@student.umw.edu.pl; 3 Department of Emergency Medical Service, Wroclaw Medical University, Bartla 5, PL 50-367 Wroclaw, Poland; patrycja.paluszkiewicz@student.umw.edu.pl; 4 Department of Internal and Occupational Diseases, Hypertension and Clinical Oncology, Wroclaw Medical University, Borowska 213, PL 50-556 Wroclaw, Poland; rafal.poreba@umw.edu.pl

**Keywords:** coronary computed tomography angiography, cor triatriatum sinister, fibroelastoma, pulmonary veins ostial anatomy

## Abstract

Coronary computed tomography angiography (CCTA) is a noninvasive examination whose main purpose is to exclude significant stenosis in the coronary arteries. The obtained computed tomography images may also provide information about other coexisting pathologies of the heart and vessels. The paper presents images of cardiac lesions in a 44-year-old hypertensive patient who underwent CCTA, based on which significant stenosis in the coronary arteries was excluded, the suspicion of a cor triatriatum sinister was confirmed and the presence of fibroelastoma and a variant of the anatomy of the pulmonary veins ostial was confirmed. To sum up, when performing CCTA, apart from the analysis of the coronary arteries, one should remember about lesions in the remaining visible anatomical structures of the heart and large vessels.

A 44-year-old Caucasian woman presented to a cardiology clinic to assess the cardiovascular consequences of arterial hypertension. The echocardiography performed during the visit described the suspicion of a cor triatriatum sinister (Figure 1A). Due to the low to intermediate risk of coronary artery disease and the echocardiographic image, it was decided to perform the diagnosis by cardiac computed tomography with assessment of the coronary arteries (coronary computed tomography angiography, CCTA).

The CCTA revealed a soft tissue septum in the left atrium (LA) that divided its two similarly sized secondary cavities (proper and accessory atrium) (Figure 1B,C). The septum had an oblique course in coronal images, from right bottom to left upper; in sagittal images, a vertical course; in axial images, an oblique course, from right anterior to left posterior. Two right pulmonary veins and a common left pulmonary vein drain into the accessory atrium (Figure 1E–G). The left atrial appendage ostium was in the proper atrium. Between the proper atrium and the left ventricle, there was a mitral valve. No atrial septal continuity defect was observed. The radiological conclusion was confirmation of the diagnosis of cor triatriatum sinister (CTS).

Moreover, significant coronary artery disease was excluded from the CCTA study. The coronary artery calcium score was 0. There was no significant stenosis in the coronary arteries: LM, LAD, LCx and RCA (Figure 2A–C).

No aortic or mitral valve calcifications were found in the CCTA. There was no pericardial fluid and no calcification and/or thickening in the pericardial laminae. The aortic valve was morphologically and functionally tricuspid. In contact with the noncoronary cusp, on the left ventricular outflow tract, an oval accessory structure of 0.5 × 0.4 cm was visualized. The CCTA image of the lesion was highly suggestive of fibroelastoma (Figure 2D–F).

The heart cavities were of normal size, and the left ventricular myocardium was of normal thickness. The topography and width of the main arteries were typical. The variant of pulmonary veins ostial anatomy was found. The presence of two right pulmonary veins (right superior pulmonary vein and right inferior pulmonary vein) and one left pulmonary vein (left common pulmonary vein) were found (Figure 2G,H). The anatomical systemic vein ostial anatomy was visualized.

The CCTA showed normal left ventricular systolic function. Left ventricular ejection fraction (LVEF) was 68% (Figure 2I).

Other parameters of left ventricular function were as presented in Table 1.

The pathogenesis of cor triatriatum sinister is not clear. Few classifications of cor triatriatum have been established, which were suggested by Loeffler, Lam and Marin-Garcia [1,2,3]. The presented case can be classified via Lam classification as class A due to a lack of atrial septal defect.

In cor triatriatum imaging, advanced imaging techniques such as computed tomography and magnetic resonance imaging are very useful. Both techniques can help to distinguish cor triatriatum from mitral stenosis, dilatation of the coronary sinus associated with anomalous pulmonary venous return or persistent left SVC and supravalvular mitral ring. The advantage of computed tomography is the possibility of simultaneous assessment of the coronary arteries, the disadvantage of exposure to ionizing radiation. Magnetic resonance imaging, using flow sequences, allows the assessment of mitral flow from the atypical left atrium to the left ventricle. With the help of MRI cinematographic sequences, it is possible to assess the mobility of the septum separating the atrium more precisely during the heart cycle and to assess the left ventricular ejection fraction most accurately. In the case of the coexistence of additional structures, CMR morphological sequences also enable a more accurate assessment of their tissue characteristics [4].

Cor triatriatum may be asymptomatic but may also be associated with an increased risk of atrial fibrillation and LA clots. In the absence of atrial fibrillation, LA clots and pulmonary vein dilation, cor triatriatum is an incidental symptom and, especially in the elderly, does not require treatment. However, if symptoms of atrial fibrillation occur, surgical correction may be considered. The corrections are made with a good clinical outcome [5].

Considering the CCT images and the clinical condition of the patient, it was decided to leave the patient under further observation, with an appointment for further follow-up visits.

## Figures and Tables

**Figure 1:**
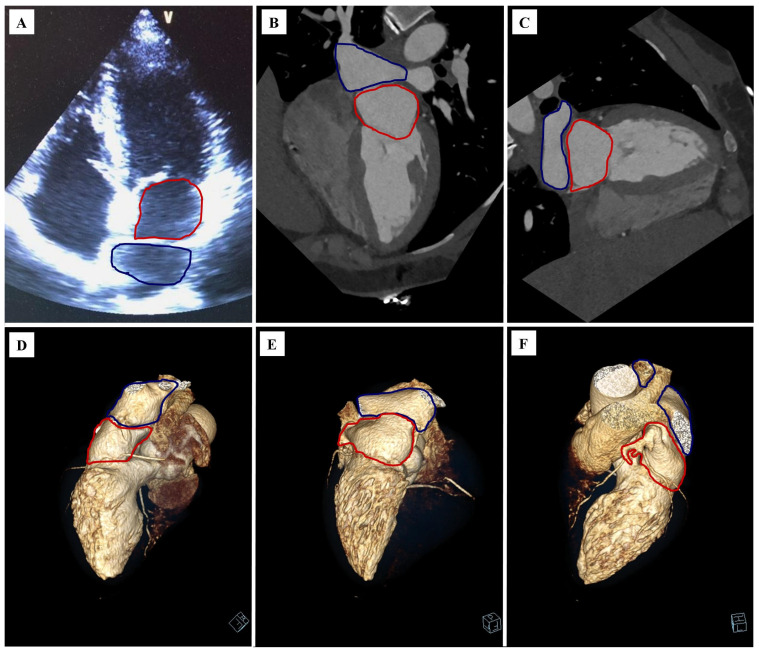
The cor triatriatum sinister. The proper atrium is marked with a red outline, while the accessory atrium is marked with a blue outline: (**A**) echocardiography, long axis, four-chamber projection; (**B**) cardiac computer tomography angiography (CCTA), multiplanar reconstruction (MPR), long axis, four-chamber projection; (**C**) CCTA, MPR reconstruction, long axis, two-chamber projection; (**D**) CCTA, volume rendering technique (VRT) reconstruction, right side view; (**E**) CCTA, VRT reconstruction, posterior view; (**F**) CCTA, VRT reconstruction, left side view.

**Figure 2:**
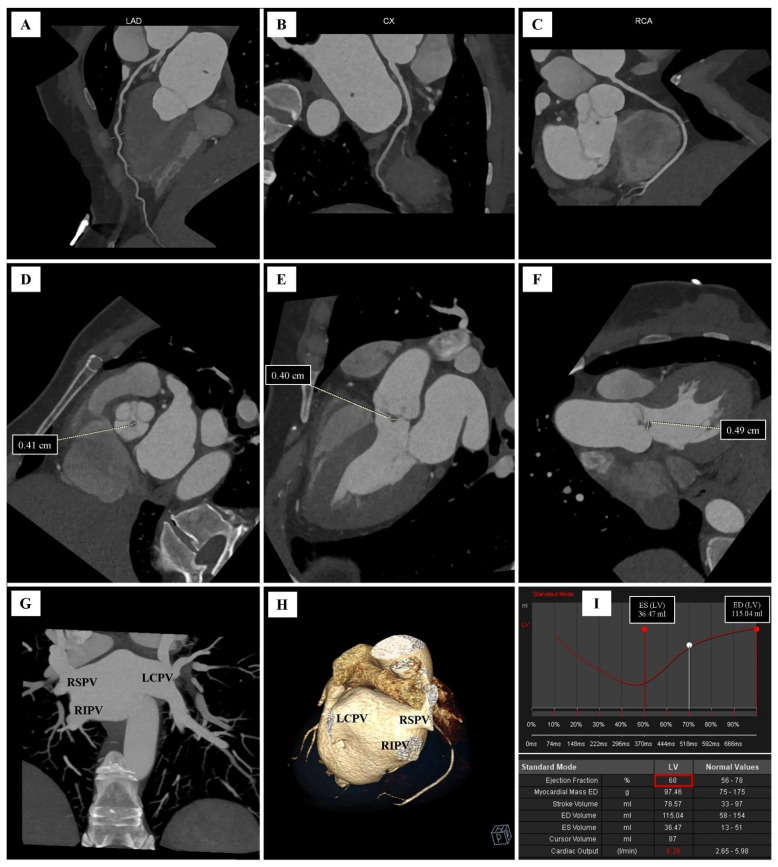
Cardiac computer tomography angiography (CCTA): (**A**) curved planar reconstruction (CPR), left anterior descending artery (LAD); (**B**) CPR reconstruction, left circumflex artery (LCx); (**C**) CPR reconstruction, right coronary artery (RCA); (**D**) multiplanar reconstruction (MPR); oblique view, parallel to the aortic annulus; dimensioning the maximum diameter of a fibroelastoma; (**E**) MPR reconstruction, long axis, three−chamber projection, dimensioning the maximum diameter of a fibroelastoma; (**F**) MPR reconstruction; long axis, parallel to the two−chamber projection; dimensioning the maximum diameter of a fibroelastoma; (**G**) maximum intensity projection (MIP) reconstruction; oblique view; right superior pulmonary vein (RSPV), right inferior pulmonary vein (RIPV) and left common pulmonary vein (LCPV) leading to the left atrium; (**H**) volume rendering technique (VRT) reconstruction; RSPV, RIPV and LCPV leading to the left atrium; (**I**) left ventricular functional assessment.

**Table 1:** Parameters of left ventricular function in coronary computed tomography angiography.

Parameter	Value
Left ventricular mass (LVM)	97.46 g
End-diastolic volume (EDV)	115.04 mL
End-systolic volume (ESV)	36.47 mL
Stroke volume (SV)	78.57 mL
Ejection fraction (EF)	68%

## Data Availability

Not applicable.
